# Nonspecific changes in clinical laboratory indicators in unselected terminally ill patients and a model to predict survival time based on a prospective observational study

**DOI:** 10.1186/1479-5876-12-78

**Published:** 2014-03-22

**Authors:** Liu Hui, Liu Qigui, Ren Sashuang, Liu Xiliang, Luan Guihong

**Affiliations:** 1 College of Medical Laboratory, Dalian Medical University, Dalian 116044, China; 2 Department of Hygienic Statistics, Dalian Medical University, Dalian 116044, China

**Keywords:** Critical care medicine, Survival time, Laboratory medicine

## Abstract

**Background:**

The clinical prediction of survival is among the most challenging tasks because it refers to the process whereby the medical team assimilates clinical data using subjective methods. The purpose of this prospective observational study was to develop a model for evaluating survival time using objective laboratory parameters.

**Methods:**

Albumin (ALB), creatinine (CRE), C-reactive protein (CRP) and the neutrophilic leukocyte count (NEU) were measured using automated analysers. A total of 177 subjects with any one positive item of 4 items were included in the study. Age on the observation date and date of death were recorded.

**Results:**

ALB, CRE, CRP and the NEU were all significant predictors of survival time (p < 0.05). The median survival time of patients with anyone of the 4 items positive would be over 1 year; if any 2 items were positive, the median survival time was approximately 1 year; if any 3 items were positive, the median survival time was approximately 4 months and if 4 items were positive, the median survival time was approximately 20 days.

**Conclusions:**

This study suggests that a model using ALB, CRE, CRP and the NEU is potentially useful in the objective evaluation of survival time in terminally ill patients.

## Background

The clinical prediction of survival in patients has the potential for significant impact on a variety of treatment decisions. Correct survival estimates can help to prevent inappropriate therapies, thereby improving the quality of end-of-life care. In addition, the ability to determine an accurate prognosis can have a major impact on supportive care for patients and their families and can facilitate timely referral for hospice care. The ability to determine prognosis is also important for the direction of research programmes and for the prioritisation of health care resources.

The clinical prediction of survival is among the most challenging tasks that a physician can face [1-3]. It refers to the process whereby the medical team assimilates clinical data using informal and subjective methods [4-6]. The nature of this approach tends to preclude precise specification.

Modern medicine, particularly medical research, requires a reliance on objective laboratory testing rather than experience and judgment alone for the diagnosis of disease. Laboratory test indicators have sensitive, accurate and quantitative characteristics. In addition to their ability to provide a reliable and objective basis for screening and diagnosis, they can also measure disease severity [7-9]. Therefore, nonspecific changes in laboratory parameters in terminally ill patients could be considered key prognostic factors in the evaluation of survival prediction in these patients. A recent study on this topic involves in biomarker profiling by nuclear magnetic resonance spectroscopy for the prediction of all-cause mortality [10]. Our study was designed to perform a quantitative analysis on changes in routine laboratory parameters and, furthermore, to explore an effective model for the evaluation of death risk using sensitive parameters that could be specifically responsive in the terminally ill. It is potentially possible to provide a more simple and precise method for the prediction of survival time in clinical application.

## Subjects and methods

### Subjects

The subjects were collected in five batches from five hospitals (First Hospital of Dalian Medical University; Second Hospital of Dalian Medical University; Central Hospital of Dalian; Hospital of Dalian University; Third Hospital of Dalian) in the city of Dalian, China, from May 2010 to May 2012. A total of 283 subjects were included in the study (155 males, 128 females; mean age 65.7 ± 14.2 years), of whom 156 were selected in an intensive care unit and 127 were selected on the ward. The beginning of observation was at collection of the blood sample. Age on the observation date and medical record number were recorded to examine more information before follow up; date of death or survival status was recorded after one year. The data regarding death and survival were obtained from the medical records and residence queries. Subjects who were lost to follow up were recorded as censored cases.

The experiments were conducted in accordance with the Declaration of Helsinki. The blood samples taken were part of the usual care of the subjects and were not taken for research purposes alone. The Institutional Ethics Committee of Dalian Medical University approved the study and waived the need for written informed consent from the participants due to the observational nature of the study.

### Blood sampling and blood analyses

Blood was drawn from the antecubital vein at 07.00 am into two vacuum tubes; one tube contained an anticoagulant and was used for blood cell counts, and the other was used for the determination of biochemical indices. Measurement of biochemical parameters, which included albumin (ALB, 40-55 g/L), aspartate aminotransferase (AST, <40 U/L) and creatinine (CRE, 45-105 μmol/L), was performed using an automatic biochemistry analyser (Hitachi7600-110). The level of high-sensitivity C-reactive protein (CRP, <8.2 mg/L) was determined by nephelometry, which involved performing a latex particle-enhanced immunoassay using a Beckman Access IMMAGE Immunochemistry System. The assay was sensitive enough to detect 0.02 mg/L of serum CRP. Measurement of blood cell counts, which included neutrophilic leukocyte (NEU, 3.5-9.5 × 10^9^/L), lymphocyte (LYM, 1.1-3.2 × 10^9^/L), red blood cell (RBC, 3.8-5.8 × 10^12^/L), platelet (PLT, 125-350 × 10^9^/L), and haematoglobin (HB, 115-175 g/L) were determined using an automated haematology analyser (Sysmex 2100; Japan). The experimental investigations were performed in the clinical laboratory of our University Hospital, using standard commercial reagent kits.

### Statistical analysis and establishment of the model

The relationships of survival time with laboratory parameters were assessed by Spearman correlation. Cox regression analysis was used to assess the dead risk from laboratory parameters and was weighted by age (alpha = 0.05, two-tailed test).

The model for evaluating survival time was created using life table analysis. Prior to analysis, the abovementioned indicators were transformed into qualitative indicators based on the reference ranges; values above the upper limit were designated as positive for AST, CRE, CRP and NEU, and values below the lower limit were designated as positive for ALB, LYM, RBC, HB, PLT. The calculations were performed using SPSS software for Windows (Chicago, IL, USA).

## Results

Correlations of survival time with laboratory parameters are shown in Table 1. ALB, CRP, LYM and NEU were chosen as predictors of survival time according to correlation coefficient. The 223 subjects with anyone positive item of these 4 items were allowed to enter the prospective observational study. Cox regression analysis was used to assess the predictors, as shown in Table 2. The LYM were not significant predictors of survival time (*p* > 0.05).

**Table 1:** Correlations between survival time and laboratory parameters

**Item**	**r**	**p**	**Item**	**r**	**p**
NEU	-0.422	<0.0001	ALB	0.455	<0.0001
LYM	0.234	<0.0001	AST	-0.308	<0.0001
RBC	0.104	0.081	CRE	-0.028	0.643
HB	0.186	0.002	CRP	-0.397	<0.0001
PLT	0.107	0.073	-	-	-

**Table 2:** Cox regression analysis of dead risk from laboratory parameters (ALB, CRP, LYM and NEU)

**Item**	**B**	**SE**	**p**	**Exp (B)**	**95% CI for Exp (B)**
ALB	-0.101	0.030	0.001	0.904	0.852~0.958
CRP	0.011	0.005	0.018	1.011	1.002~1.020
LYM	-0.184	0.202	0.363	0.832	0.560~1.236
NEU	0.159	0.037	<0.0001	1.172	1.091~1.259

ALB, NEU, CRP and CRE were chosen as predictors of survival time according to correlation coefficient as well as mechanisms. The 177 subjects with anyone positive item of 4 items (ALB, CRE, CRP and NEU) were allowed to enter the prospective observational study. Cox regression analysis revealed that ALB, NEU, CRP and the CRE were all significant predictors of survival time (*p* < 0.05), as shown in Table 3. The levels of ALB, NEU, CRP and CRE in different dead time are shown in Table 4.

**Table 3:** Cox regression analysis of dead risk from laboratory parameters (ALB, CRE, CRP and NEU)

**Item**	**B**	**SE**	**p**	**Exp (B)**	**95% CI for Exp (B)**
ALB	-0.091	0.032	0.005	0.913	0.857~0.972
CRE	0.003	0.001	0.031	1.003	1.000~1.005
CRP	0.010	0.005	0.028	1.010	1.001~1.019
NEU	0.135	0.036	<0.0001	1.145	1.067~1.228

**Table 4:** Levels of ALB, CRE, and CRP and the NEU in different dead time

**Dead groups (days)**	**Quartile (25**^**th**^**, 50**^**th**^**, 75**^**th**^**)**			
	**ALB**	**CRE**	**CRP**	**NEU**
0~	25.3, 29.8, 34.4	72.3, 115.1, 190.5	6.7, 19.6, 27.0	5.3, 8.7, 13.3
90~	32.3, 33.3, 35.1	63.9, 83.3, 130.3	5.0, 29.7, 58.8	3.9, 5.4, 9.2
180~	31.2, 34.4, 37.7	77.5, 87.3, 95.8	4.4, 24.3, 57.8	2.7, 3.7, 7.3
270~	26.3,32.7, 39.4	80.0, 106.9, 134.7	1.5, 3.2, 47.1	3.8, 8.5, 9.3
>360	32.5, 35.7, 39.6	84.8, 107.6, 120.8	1.3, 4.8, 15.1	3.1, 4.5, 6.4

A total of 177 subjects were included in this study (114 males and 63 females; mean age 66.5 ± 13.6 years). The main diseases were cardiovascular and cerebrovascular disease (20.3%), chronic obstructive pulmonary disease (9.6%), various types of tumours (44.1%) and other diseases not including accidental injuries (26.0%). After one year, a total of 93 subjects died including 27 patients with cardiovascular and cerebrovascular disease (29.0%), 6 patients with chronic obstructive pulmonary disease (6.5%), 46 patients with various types of tumours (49.5%) and 14 patients with other diseases (15.0%).

**Figure 1:**
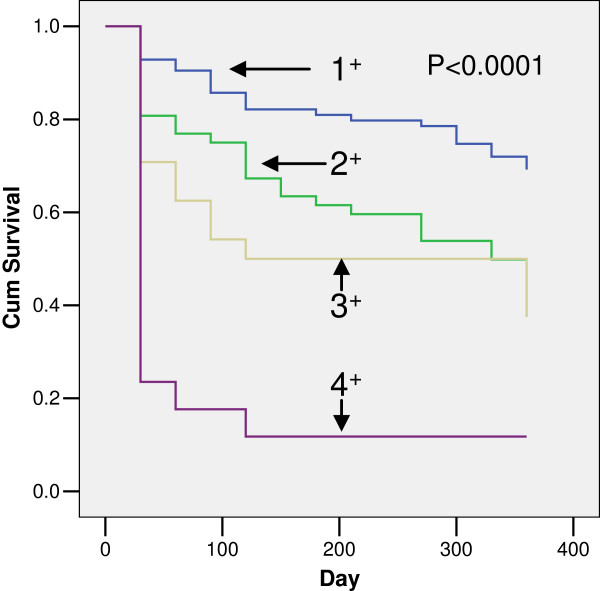
**Difference in survival times of patients with one (1**^**+**^**), two (2**^**+**^**), three (3**^**+**^**) or four (4**^**+**^**) positive laboratory indicators among ALB, CRE, CRP and NEU.**

Table 5 presents the establishment of a predictive model of the survival time of critically ill patients and an analysis of the results. The median survival times of patients with one, two, three or four (all) positive laboratory indicators were as follows: one, over 360 day; two, 328.9 days (approximately 1 year); three, 120.0 days (approximately 4 months); and four, 19.6 days. There was a significant difference in the survival times of patients with one, two, three or four of four positive items, as shown in Figure 1.

**Table 5:** The original data and establishment of a predictive model of survival time

**Observed time (days)**	**Number (death + censored)**				**Total number**
	**1**^**+**^	**2**^**+**^	**3**^**+**^	**4**^**+**^	
0~	6 + 0	10 + 0	7 + 0	13 + 0	36
30~	2 + 0	2 + 0	2 + 0	1 + 0	7
60~	4 + 0	1 + 0	2 + 0	1 + 0	8
90~	3 + 0	4 + 0	1 + 0	0 + 0	8
120~	0 + 0	2 + 0	0 + 0	0 + 0	2
150~	1 + 0	1 + 0	0 + 0	0 + 0	2
180~	1 + 0	1 + 0	0 + 0	0 + 0	2
210~	0 + 0	0 + 0	0 + 0	0 + 0	0
240~	1 + 0	3 + 0	0 + 0	0 + 0	4
270~	3 + 9	0 + 1	0 + 0	0 + 0	13
300~	2 + 0	2 + 0	0 + 0	0 + 0	4
330~	2 + 0	2 + 0	3 + 0	0 + 0	7
>360	0 + 50	0 + 23	0 + 9	0 + 2	84
Total	25 + 59 = 84	28 + 24 = 52	15 + 9 = 24	15 + 2 = 17	177
MST (days)	>360.0	328.9	120.0	19.6	-

Patients with one (1^+^), two (2^+^), three (3^+^) or four (4^+^) positive laboratory indicators among ALB, CRE, CRP and NEU.

MTS: Median survival time.

## Discussion

The hypothesis for this study was that any test results related to death are likely occur within one year of death and are unlikely to be related to a death occurring after one year. Therefore, we conducted a one-year prospective observational study in a group of critically ill patients based on blood parameters.

For the routine laboratory test indicators, the research basis are usually solid and under the good quality control; hence, the prognostic value of routine clinical and biochemical parameters was evaluated in this study. ALB, CRP, LYM and NEU should be chosen as predictors of survival time according to correlation coefficient; however, these 4 items were considered as an integer for assessment using cox regression analysis, the LYM showed no significance as determined by a p value = 0.363; whereby other parameters should be selected for the analysis.

Natural death for the majority people can be considered as a process of loss of organ function. Blood biochemical studies are used in the assessment of organ function. The identification of blood laboratory parameters in terminally ill patients is essential to our understanding of the impairment and restorative processes that occur during the end of life and the functional consequences of hypoergia. Therefore CRE, which reflects renal function, was chosen as a predictor in spite of relative small correlation coefficient of CRE.

The laboratory parameters that appeared responsive to death included ALB, CRE, CRP and the NEU, and the trend of the response was consistent with the pathological changes [11-13]. Therefore, these laboratory parameters can be considered a quantitative evaluation of holistic function. Cox regression analysis revealed that ALB, NEU, CRP and the CRE were all significant predictors of survival time (*p* < 0.05).

The critically ill patients in this study had been diagnosed with tumours, cardiovascular and cerebrovascular diseases and chronic obstructive pulmonary disease. In China, these three diseases account for over 70% of all-cause mortality [14], and they accounted for 74% (131/177) of disease in this observational group and 85% (79/93) of disease in dead subjects. Although the different types and severity of disease as well as ageing may affect survival times, we believe that laboratory parameters can reflect the relation of these factors with survival time, thus simplifying complex issues. The results from this study demonstrate significant changes in 4 laboratory indicators. These findings are consistent with our previous study [7-9], indicating that these laboratory parameters could have potential in the prediction of survival in critically ill patients.

ALB reflects liver function, CRE reflects renal function, and CRP and the NEU reflect systemic inflammation. Abnormal changes in two or more indicators can be considered as nonspecific systemic changes, which are associated with death, whereas abnormal changes in a single indicator may be specific. We transformed the measurement data into qualitative data by taking the upper or lower limit of the reference range for each indicator as the cutoff value. As data transformation was conducted according to the reference range of each item, theoretically, the reference range was determined by 95% of the population. Therefore, the positivity rate in the normal population was less than 5% in a single index. According to the combination principle, the probability of a normal individual with two or more positive factors among the 4 laboratory indicators is rare, whereas the positivity rate for any one of four items is theoretically 5% × 4 = 20% in a healthy population and may be not related to the frequency of mortality. Therefore, subjects with only one positive indictor were included as control group and patients who demonstrated two or more two positive laboratory indicators were included as observed groups.

The results indicate that if a patient presents any 1 of 4 items as positive, the expected survival time (50% survival probability) will be over 1 year; if any 2 items are positive, the expected survival time will be approximately 1 year (328.9 days); if any 3 items are positive, the expected survival time will be approximately 4 months (120.0 days); and if 4 items are positive, the expected survival time will be approximately 20 days. This study suggests that a model using ALB, CRE, CRP and the NEU is potentially useful in the objective evaluation of survival time in terminally ill patients.

Although the accurate prediction of survival is essential for palliative care and several clinical tools predicting survival time exist [15-17], these approaches are not considered appropriate [18]. This largely reflects the diverse and complex causes of death. Clearly, our findings are helpful for the establishment of a model using biomarkers to improve survival prediction. Further studies are warranted to develop a more available model for the accurate prediction of survival with routine laboratory parameters.

## Conclusions

Despite the complex causes of death, our results emphasise that ALB, CRE, CRP and the NEU may reflect the relation of these complex factors with survival times and could be considered as key prognostic factors. A model without complex mathematical calculation should be suggested for predicting survival time based on these routine laboratory parameters.

## Abbreviations

ALB: Serum albumin; AST: Aspartate aminotransferase; CRE: Serum creatinine; CRP: C-reactive protein; HB: Haematoglobin; LYM: Lymphocyte; NEU: Neutrophilic leukocyte; PLT: Platelet; RBC: Red blood cell.

## Competing interests

The authors declare that they have no competing interests.

## Authors’ contributions

LH participated in the design of the study, performed the statistical analysis and wrote the paper. LQ performed the statistical analysis and critically revised the manuscript. RS, LX and LG performed the data acquisition and experiments. All authors read and approved the final manuscript.
